# Dual-Branch Multi-Perspective Modulation Network for Efficient Infrared Image Super-Resolution

**DOI:** 10.3390/s26154928

**Published:** 2026-08-04

**Authors:** Zepeng Liu, Duanyang Zhang, Ruimin Qi, Guodong Zhang, Yizong Wang, Lizhi Liu

**Affiliations:** 1 School of Information Engineering, Xinjiang Institute of Technology, Akesu 843100, China; 2022230@xjit.edu.cn (D.Z.); 2023022@xjit.edu.cn (R.Q.); 2023021@xjit.edu.cn (G.Z.); 2019109@xjit.edu.cn (Y.W.); 2 School of Information and Communication Engineering, University of Electronic Science and Technology of China, Chengdu 611730, China; 3 School of Electrical Engineering, Liaoning University of Technology, Jinzhou 121001, China; liulizhi@emails.imau.edu.cn

**Keywords:** infrared image super-resolution, self-attention, multi-perspective, cross modulation

## Abstract

Transformer-based super-resolution methods achieve competitive performance by capturing non-local information through self-attention. Nevertheless, the computation of the self-attention introduces heavy computational overhead, and its inherent low-pass characteristic restricts the learning of local details. To address these problems, we propose an effective dual-branch multi-perspective modulation network (DMMN) for efficient infrared image super-resolution. Specifically, we design a multi-scale feature modulation enhancement unit (MFMEU) to capture cross-scale spatial features and a frequency-domain cross-correlation patch modulation unit (FCPMU) to explore global feature representations. We further develop an efficient bidirectional cross modulation unit (BCMU) to promote feature interaction between outputs of MFMEU and FCPMU. Extensive experimental results verify that DMMN achieves a competitive trade-off between reconstruction accuracy and computational efficiency. For instance, compared with the ×4 SRFormer-light, the proposed DMMN obtains an average gain of 0.08 dB in PSNR across five public test datasets, runs 2.7× faster, and uses only about 24% of the FLOPs.

## 1. Introduction

Infrared image super-resolution (IRSR) aims to reconstruct high-resolution (HR) outputs from degraded low-resolution (LR) images. It is widely used in real-time key scene tasks, such as military reconnaissance [1], security surveillance [2] and night-vision navigation [3,4]. However, due to the physical characteristics of infrared imaging equipment, the obtained images usually have low resolution, poor contrast and blurred details, which seriously limits the performance of downstream tasks. Therefore, it is crucial to efficiently reconstruct high-quality infrared images with limited computing resources.

Convolutional neural network (CNN)-based [5,6,7,8,9,10,11] methods achieve favorable reconstruction performance through hierarchical feature extraction in super-resolution (SR). However, these methods limit their ability to model non-local dependencies because of their fixed receptive fields. To remedy this bottleneck, CNN-based works [7,8,12] expand network depth and width for richer feature representation, e.g., RCAN builds over 400 stacked layers with 15.59 M parameters. Even though this design improves reconstruction accuracy, such architectures incur substantial computational costs, hindering their practical deployment in resource-constrained scenarios. Visual Transformer (ViT)-based SR methods [13,14,15,16,17,18,19] model non-local features through self-attention (SA). However, the implementation of SA requires massive computing resources and memory. To alleviate the computational burden, windowed SA [13,20], transposed attention [21], and hierarchical aggregation [16] are developed to reduce computational cost. Moreover, recent studies confirm ViT architectures act as implicit low-pass filters, yielding smooth reconstruction results [22,23].

Deep learning-driven IRSR approaches [24,25,26,27,28,29,30,31] have achieved notable progress. However, these methods face two coexisting bottlenecks for achieving efficient IRSR. On the one hand, many are tailored for specific IRSR scenarios, such as infrared small target [32], domain adaptation [26], or particular infrared physical characteristics [27], lacking a unified modeling framework. On the other hand, high-fidelity methods [28,29,31,33] incur substantial computational overhead, making them difficult to deploy on resource-constrained infrared devices. Therefore, it is urgent to develop an IRSR that balances reconstruction accuracy and computational efficiency.

**Figure 1:**
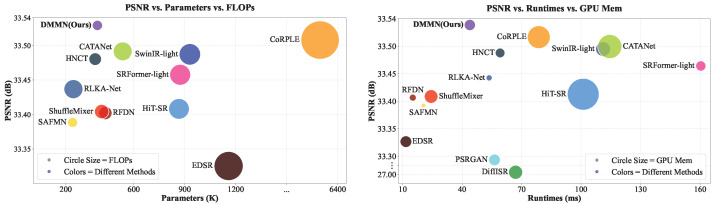
Comparison of model complexity and reconstruction performance among lightweight super-resolution methods for the ×4 SR task on the CVC-09 [34] infrared dataset. The left subplot illustrates the relationship between PSNR and computational cost, where computational cost encompasses the number of parameters and FLOPs, and the size of each circle indicates the FLOPs magnitude. The right subplot shows the relationship between PSNR, inference time and GPU memory usage, with the size of each circle representing GPU memory consumption. The proposed DMMN effectively balances computational overhead (i.e., parameters, FLOPs, runtime and GPU Mem) with reconstruction performance (i.e., PSNR).

To address the above limitations, we propose a dual-branch multi-perspective modulation network (DMMN) for efficient IRSR. Specifically, we propose a multi-scale feature modulation enhancement unit (MFMEU) to capture local and non-local features, and a frequency-domain cross-correlation patch modulation unit (FCPMU) to explore global information. We further develop a bidirectional cross modulation unit (BCMU) to modulate complementary information from MFMEU and FCPMU across both channel and spatial dimensions. Extensive results demonstrate that the proposed DMMN achieves a favorable balance between reconstruction accuracy and computational overhead (see Figure 1). Therefore, our DMMN is expected to promote the practical application of IRSR in key areas such as military reconnaissance, security monitoring and unmanned systems. To further clarify the research gaps of existing super-resolution methods, we review relevant visible and infrared image super-resolution literature in the following section.

The main contributions of this paper are summarized as follows:

- We propose an efficient MFMEU for capturing local and non-local features and a lightweight FCPMU for exploring global information.
- We design a BCMU to sequentially modulate complementary information derived from MFMEU and FCPMU across both channel and spatial dimensions.
- We conduct extensive experiments across five benchmark datasets, and the results demonstrate that the proposed DMMN achieves a favorable balance between reconstruction accuracy and computational efficiency.

## 2. Related Work

### 2.1. Visible Image Super-Resolution

CNN-based image super-resolution. SRCNN establishes the first end-to-end deep learning paradigm for single-image super-resolution. Follow-up architectures including VDSR [12], DRCN [35] and DRRN [36] introduced residual connections and recursive block designs to speed up training convergence and boost reconstruction fidelity. To strengthen feature representation capacity, subsequent works [7,8] continuously expanded network width and depth. EDSR [7] expands to 32 layers with 43 million parameters, while RCAN [8] integrates channel attention and stacks over 400 layers. However, their massive parameter and computational costs hinder practical deployment in resource-constrained scenarios.

To mitigate computational overhead, numerous CNN-based methods [6,9,10,11] have been developed to balance reconstruction performance and computational efficiency. FSRCNN [6] and ESPCN [37] improve computational efficiency by adopting post-upsampling strategy, while CARN [38] leverages group convolution and cascaded feature aggregation for efficient SR. IDN [39] and IMDN [40] adopt an information distillation strategy to achieve an effective trade-off between memory and real-time performance. RFDN [9] further enhances this paradigm and won the NTIRE 2022 Efficient SR Challenge. ShuffleMixer [41] utilizes large kernels combined with channel splitting and shuffling to reduce FLOPs. Recent works [10,11] introduce dynamic feature modulation to capture deep features for reconstruction. For instance, SAFMN [10] develops spatial adaptive modulation within a ViT-like architecture, and SMFANet [11] captures both local and non-local features through a dual-branch design. Despite their ability to achieve promising performance with low computational cost, these methods still lack sufficient global modeling capacity due to fixed convolution kernels and single-scale feature extraction patterns. In contrast, we propose an FCPMU to explore global information from a frequency-domain perspective.

ViT-based image super-resolution. Vision Transformer (ViT) [42] achieves competitive restoration performance by capturing long-range feature correlations via SA [43]. IPT first pioneers pre-trained Transformer backbones for low-level vision and built a universal modeling pipeline for diverse image restoration tasks [44]. However, the SA mechanism requires high computational resources, so various attention variants are proposed to reduce the computational overhead. SwinIR [13] adopts windowed SA to reduce computational cost, while Restormer [21] refines SA and feed-forward modules to facilitate interaction between global and local features. Subsequent SA variants [21] further enhance reconstruction fidelity. For instance, HAT [17] combines channel attention with window-based and overlapping cross-attention to activate more effective pixels, whereas DAT [45] and DRCT [46] enhance feature aggregation and stabilize deep network propagation through dual aggregation and dense residual connections. To strike a better balance between performance and efficiency, lightweight Transformer models have emerged as a research focus. ELAN [20] achieves efficient long-range modeling by employing shift convolution and grouped multi-scale SA, substantially reducing redundant computation. NGswin [47] introduces N-Gram context to expand window interaction ranges, thereby enhancing global modeling within a lightweight framework. HiT-SR [16] and CATANet [15] further reduce complexity through hierarchical window strategies and content-aware token aggregation with cross-attention, respectively. Meanwhile, methods such as SPIN [48] and MAT [19] control computational overhead via super-pixel grouping and multi-range dilation attention. Despite these advances, these methods still require higher computational cost to explore global feature dependencies for SR. Moreover, recent studies reveal ViTs tend to learn low-frequency components to obtain smooth reconstruction results. To address the above issues, we propose a lightweight MFMEU to capture local and non-local features and an efficient FCPMU to explore global information. The above lightweight visible SR methods cannot be directly applied to infrared imaging due to the unique low-contrast, blurry texture characteristics of infrared data. We next systematically summarize dedicated IRSR research.

### 2.2. Infrared Image Super-Resolution

Deep learning has made significant progress in IRSR due to its powerful automatic feature extraction. TEN [24] pioneers end-to-end CNN-based mapping, while IE-CGAN [49] leverages conditional generative adversarial networks with residual structures and visible-light transfer to mitigate data scarcity. Subsequent works further advanced this direction. PSRGAN [25] adopts a dual-path architecture for joint infrared and visible feature extraction combined with transfer learning, and DASRGAN [26] introduces texture- and noise-oriented domain adaptation to suppress artifacts. MoCoPnet [32] incorporates local motion and contrast priors to achieve small target reconstruction, whereas EIRSR [27] designs a row-column transformation module to better model temperature continuity and pixel correlation. Real-IISR [33] addresses real infrared coupled degradation through an autoregressive framework with thermal structure guidance. Recently, LKFormer [28], SwinIBSR [29], and DifIISR [30] further improved performance using large-kernel Transformers, infrared degradation modeling, and gradient-guided diffusion models, respectively. Despite these advances in reconstruction accuracy, most methods are tailored for specific scenarios, resulting in the absence of a unified IRSR modeling framework. Additionally, their high computational overhead and memory usage hinder deployment on resource-constrained infrared imaging devices.

To reduce the computational burden, many methods [31,50,51,52] aim to achieve a balance between performance and efficiency. RLKA-Net [50] introduces recursive large kernel attention to deliver compact non-local modeling with fewer parameters, while LISN [51] adopts shift operations paired with lightweight attention modules to significantly reduce computational overhead. LIRSRN [53] embeds joint spatial-frequency processing into enhanced attention units for precise restoration of fine textures and edge contours. CoRPLE [31] leverages contourlet transformation for compact feature modeling, while IRSRMamba [52] realizes efficient feature extraction by integrating Mamba and wavelet modules. Mansoor performs evaluation across multiple independent datasets and difficult real-world samples for efficient medical endoscopic SR [54]. Despite these advances, most methods sacrifice high-frequency texture reconstruction to pursue efficiency.

## 3. Proposed Method

**Figure 2:**
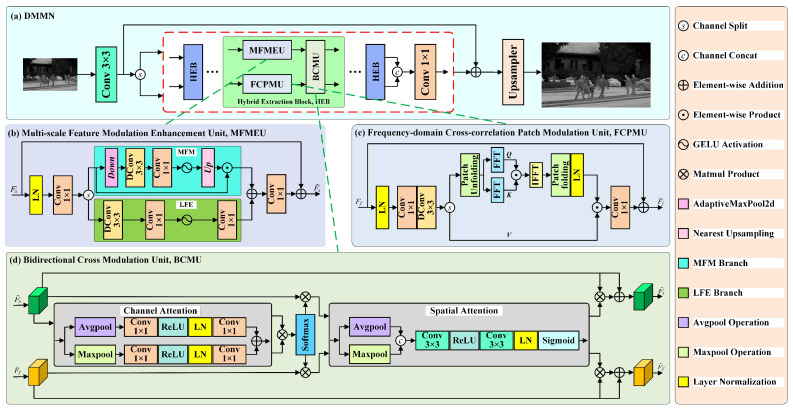
Network architecture of the proposed DMMN. The proposed DMMN comprises a shallow features layer, multiple stacked hybrid extraction blocks (HEBs), and a lightweight upsampler module. Each HEB consists of a multi-scale feature modulation enhancement unit (MFMEU), a frequency-domain cross-correlation patch modulation unit (FCPMU) and a bidirectional cross modulation unit (BCMU).

Figure 2 illustrates the overall architecture of the proposed DMMN. We first extract shallow feature embeddings for LR inputs via a 3×3 convolutional layer. These shallow features are then fed into stacked hybrid extraction blocks (HEBs) to produce deep representative features. Specially, each HEB contains a multi-scale feature modulation enhancement unit (MFMEU) for capturing local and non-local features and a frequency-domain cross-correlation patch modulation unit (FCPMU) for exploring global information in parallel. Furthermore, we develop a bidirectional cross modulation unit (BCMU) for facilitating information interaction between MFMEU and FCPMU outputs. After passing through the HEBs, the latent features undergo channel concatenation and a 1×1 convolutional layer to aggregate these complementary feature representations. The fused representations are delivered to a lightweight upsampler module, which comprises a 3×3 convolution and a PixelShuffle layer [10,11], to generate the HR outputs. A global residual connection is introduced before the upsampler module to effectively propagate global structural information and mitigate gradient vanishing during training.

Following previous work [10,11,41], we adopt a joint loss function combining mean absolute error (MAE) loss and fast Fourier transform (FFT) frequency loss for parameter optimization, defined as:

(1) $$\begin{array}{cc} \; & L={∥I_{SR}-I_{HR}∥}_{1}+\lambda {∥F\left(I_{SR}\right)-F\left(I_{HR}\right)∥}_{1} \end{array}$$

where $I_{HR}$ and $I_{SR}$ denote the ground-truth and reconstructed high-resolution infrared images, respectively. ${∥\cdot ∥}_{1}$ represents the $L_{1}$-Norm, and $F\left(\cdot \right)$ stands for the FFT operation. The weighting coefficient λ balances the impact of the two loss components, which is empirically assigned to 0.5 according to the ablation results.

### 3.1. Multi-Scale Feature Modulation Enhancement Unit

Exploring non-local and local features at the same time is critical for IRSR reconstruction. Existing ViT-based SR approaches [13,14,17,20] exploit various SA schemes to capture long-range correlations and attain promising restoration quality. Nevertheless, the SA mechanism and its variants suffer from high computational overhead and weak local detail modeling capacity. This deficiency derives from their inherent low-pass filtering characteristic, which inclines networks to prioritize low-frequency components [22,23]. To tackle this issue, we design a MFMEU, which adopts a dual-parallel architecture consisting of a multi-scale feature modulation (MFM) branch and a local feature enhancement (LFE) branch. The MFM branch excavates non-local information via downsampling and depthwise convolution, whereas the LFE branch concentrates on local feature extraction.

Given an input $F_{s}\in R^{H\times W\times C}$, we first employ a normalization layer followed by a 1×1 convolutional layer to generate projected feature $F_{p}\in R^{H\times W\times 2C}$. Next, $F_{p}$ is equally partitioned along the channel dimension into two feature tensors $X,Y\in R^{H\times W\times C}$. The above process is formulated as:

(2) $$\begin{array}{cc} \; & F_{p}=Conv_{1\times 1}\left(LN\left(F_{s}\right)\right),\\ \; & \left[X,Y\right]=Split\left(F_{p}\right), \end{array}$$

where $LN\left(\cdot \right)$ denotes the layer normalization, $Conv_{1\times 1}\left(\cdot \right)$ represents a 1×1 convolutional layer, and $Split\left(\cdot \right)$ stands for channel split. *X* and *Y* are separately fed into the MFM branch and LFE branch for feature extraction. The outputs from the two branches are then aggregated via element-wise summation, and a subsequent 1×1 convolution yields the ultimate module output. The overall procedure can be expressed as:

(3) $$\begin{array}{cc} \; & X_{m}=MFM\left(X\right),Y_{d}=LFE\left(Y\right),\\ \; & {\tilde{F}}_{s}=Conv_{1\times 1}\left(X_{m}+Y_{d}\right)+F_{s}, \end{array}$$

where ${\tilde{F}}_{s}\in R^{H\times W\times C}$ denotes the feature output of the MFMEU.

Multi-scale Feature Modulation Branch. The MFM branch is designed to explore cross-scale spatial complementary information for infrared images. By fusing multi-scale information for feature modulation, the network gains simultaneous awareness of global context and local textures, mitigating the deficiency of SA in recovering local features. Specifically, given an input $X\in R^{H\times W\times C}$, we first perform downsampling to extract low-frequency components and adopt a 3×3 depth-wise convolution to capture non-local features. A successive 1×1 followed by the GELU activation further performs nonlinear feature transformation. We then adopt nearest-neighbor upsampling to restore the feature map to its original spatial resolution. We realize cross-scale modulation via element-wise multiplication between the upsampled representation and the original input *X*. This process can be expressed as:

(4) $$\begin{array}{cc} \; & X_{m}=X⊙U\left(\phi \left(Conv_{1\times 1}\left(DWConv_{3\times 3}\left(D\left(X\right)\right)\right)\right)\right), \end{array}$$

where $D\left(\cdot \right)$ denotes adaptive max pooling with a scaling factor of 8, $DWConv_{3\times 3}\left(\cdot \right)$ represents 3×3 depth-wise convolution, $\phi \left(\cdot \right)$ refers to GELU activation, $U\left(\cdot \right)$ denotes nearest-neighbor upsampling, and ⊙ represents element-wise multiplication. $X_{m}\in R^{H\times W\times C}$ is the modulated feature output.

Local Feature Enhancement Branch. The LFE branch adopts an “expansion–activation–compression” structure to strengthen local texture representation. Given an input $Y\in R^{H\times W\times C}$, a 3×3 depth convolution first expands channel dimensions and captures local features. A successive 1×1 convolution coupled with GELU activation endows the module with nonlinear modeling capability. Another 1×1 convolution then recovers the original channel dimension and further boosts local feature responses. The entire procedure is formulated as:

(5) $$\begin{array}{cc} \; & Y_{d}=Conv_{1\times 1}\left(\phi \left(Conv_{1\times 1}\left(DWConv_{3\times 3}\left(Y\right)\right)\right)\right), \end{array}$$

where $Y_{d}\in R^{H\times W\times C}$ denotes the final local enhanced feature.

### 3.2. Frequency-Domain Cross-Correlation Patch Modulation Unit

Global information modeling is essential for reconstructing high-quality infrared images. Existing methods [14,17,18] exploit SA and its variants to capture long-range global dependencies, achieving favorable reconstruction performance. Nevertheless, such attention-based methods suffer from heavy computational costs and limit local detail modeling capacity. To address this limitation, we propose a FCPMU for efficient global feature extraction from frequency-domain perspective. Given an input feature $F_{f}\in R^{H\times W\times C}$, we first perform layer normalization, followed by a 1×1 convolution and 3×3 depth-wise convolution to generate the projected feature $F_{p}\in R^{H\times W\times 6C}$. Subsequently, we equally split $F_{p}$ along the channel dimension to yield query $F_{q}\in R^{H\times W\times 2C}$, key $F_{k}\in R^{H\times W\times 2C}$ and value $F_{v}\in R^{H\times W\times 2C}$. The above process is formulated as:

(6) $$\begin{array}{cc} \; & F_{p}=DWConv_{3\times 3}\left(Conv_{1\times 1}\left(LN\left(F_{f}\right)\right)\right),\\ \; & \left[F_{q},F_{k},F_{v}\right]=Split\left(F_{p}\right), \end{array}$$

We then unfold $F_{q}$, $F_{k}$ into non-overlapping patches with a uniform patch size of P=8. Each patch yields a dimension of P×P×2C, and the total number of patches is calculated as $N=\frac{H}{P}\cdot \frac{W}{P}$. This unfolding operation produces patch sets ${\left\{q_{i}\right\}}_{i=1}^{N}$ and ${\left\{k_{i}\right\}}_{i=1}^{N}$, where $q_{i},k_{i}\in R^{P\times P\times 2C}$. Conventional spatial SA flattens these patches into matrix forms $Q,K\in R^{N\times 2CP^{2}}$ for dot-product similarity calculation, which inevitably introduces quadratic computational complexity. The scaled dot-product attention is formulated as:

(7) $$\begin{array}{cc} \; & F_{qk}=Softmax\left(\frac{QK^{⊤}}{\sqrt{CH_{P}W_{P}}}\right), \end{array}$$

where $K^{⊤}$ denotes the transpose matrix of *K*. The matrix multiplication $QK^{⊤}$ calculates the similarity between each query and all key embeddings to generate the attention matrix. The individual elements are derived from inner-product computation:

(8) $$\begin{array}{cc} \; & {\left(QK^{⊤}\right)}_{ij}=\langle q_{i},k_{j}\rangle , \end{array}$$

As dictated by the convolution theorem, spatial-domain convolution between two signals is equivalent to element-wise multiplication in the frequency-domain. This naturally inspires a critical question: *Can we estimate attention maps efficiently via frequency-domain element-wise multiplication rather than performing expensive spatial matrix multiplication $QK^{⊤}$?*

To alleviate the computational overhead, we adopt FFT to compute the correlation between $F_{q}$ and $F_{k}$ in the frequency-domain. In this process, only real components are preserved, while numerically induced imaginary components are discarded. The resulting correlation features are subsequently normalized via layer normalization and multiplied element-wise with the value feature $F_{v}$ to achieve adaptive feature modulation. The procedure is expressed as:

(9) $$\begin{array}{cc} \; & F_{m}=LN\left(F^{-1}\left(F\left(F_{q}\right)\bar{F\left(F_{k}\right)}\right)\right)⊙F_{v}, \end{array}$$

where $F_{m}\in R^{H\times W\times 2C}$ denotes the modulated real-valued feature, $F\left(\cdot \right)$ and $F^{-1}\left(\cdot \right)$ represent the FFT and inverse FFT operations, and $\bar{F(\cdot )}$ refers to the complex conjugate operation. Finally, we use a 1×1 convolution for feature aggregation, followed by a residual connection with the input feature $F_{f}$ to produce the final module output. The process is formulated as:

(10) $$\begin{array}{cc} \; & {\tilde{F}}_{f}=F_{f}+Conv_{1\times 1}\left(F_{m}\right), \end{array}$$

where ${\tilde{F}}_{f}\in R^{H\times W\times C}$ denotes the final output of the FCPMU.

### 3.3. Bidirectional Cross Modulation Unit

Most existing methods perform feature modeling from only a single perspective [7,10,50,51,55], lacking the interaction between local and global information. Additionally, some methods [11,31,56,57] employ simple concatenation or summation for feature fusion, which cannot adequately utilize complementary information from different feature streams. To tackle these limitations, we develop a BCMU that realizes bidirectional feature interaction between MFMEU and FCPMU through sequential channel and spatial attention mechanisms. Given two input features ${\tilde{F}}_{s},{\tilde{F}}_{f}\in R^{H\times W\times C}$, we separately perform global average pooling (GAP) and global max pooling (GMP) along the spatial dimension to capture global channel-wise statistics, as formulated below:

(11) $$\begin{array}{cc} \; & {\tilde{F}}_{s}^{a}=GAP\left({\tilde{F}}_{s}\right),{\tilde{F}}_{s}^{m}=GMP\left({\tilde{F}}_{s}\right),\\ \; & {\tilde{F}}_{f}^{a}=GAP\left({\tilde{F}}_{f}\right),{\tilde{F}}_{f}^{m}=GMP\left({\tilde{F}}_{f}\right), \end{array}$$

where ${\tilde{F}}_{s}^{a},{\tilde{F}}_{s}^{m},{\tilde{F}}_{f}^{a},{\tilde{F}}_{f}^{m}\in R^{1\times 1\times C}$ denote the pooled feature representations. These pooled features are subsequently fed into a multilayer perceptron (MLP) to facilitate nonlinear mapping. The MLP sequentially adopts a 1×1 convolution for channel expansion, ReLU activation, layer normalization, and another convolution for channel compression. The procedure is formulated as:

(12) $$\begin{array}{cc} \; & {\tilde{F}}_{s}^{al}=MLP\left({\tilde{F}}_{s}^{a}\right),{\tilde{F}}_{s}^{ml}=MLP\left({\tilde{F}}_{s}^{m}\right),\\ \; & {\tilde{F}}_{f}^{al}=MLP\left({\tilde{F}}_{f}^{a}\right),{\tilde{F}}_{f}^{ml}=MLP\left({\tilde{F}}_{f}^{m}\right), \end{array}$$

where ${\tilde{F}}_{s}^{al},{\tilde{F}}_{s}^{ml},{\tilde{F}}_{f}^{al},{\tilde{F}}_{f}^{ml}\in R^{1\times 1\times C}$ denote the MLP-transformed weights. These transformed weights are aggregated through element-wise summation to generate fused weights ${\tilde{F}}_{s}^{t},{\tilde{F}}_{f}^{t}\in R^{1\times 1\times C}$. The weights ${\tilde{F}}_{s}^{t}$ and ${\tilde{F}}_{f}^{t}$ are multiplied, and the output is processed by Softmax to yield cross-channel attention weight ${\tilde{F}}_{w}$. This process is formulated as:

(13) $$\begin{array}{cc} \; & {\tilde{F}}_{s}^{t}={\tilde{F}}_{s}^{al}+{\tilde{F}}_{s}^{ml},{\tilde{F}}_{f}^{t}={\tilde{F}}_{f}^{al}+{\tilde{F}}_{f}^{ml},\\ \; & {\tilde{F}}_{w}=Softmax\left({\tilde{F}}_{s}^{t}⊗{\tilde{F}}_{f}^{t}\right), \end{array}$$

where ⊗ denotes matrix multiplication. The obtained cross-channel attention weights are multiplied element-wise with the original inputs ${\tilde{F}}_{s}$ and ${\tilde{F}}_{f}$ to yield channel-enhanced features ${\overset{ˇ}{F}}_{s},{\overset{ˇ}{F}}_{f}\in R^{H\times W\times C}$. As for spatial attention, we utilize GAP and GMP along the channel dimension on ${\overset{ˇ}{F}}_{s}$ and ${\overset{ˇ}{F}}_{f}$. This squeezes the channel dimension to capture spatial statistics and generates pooled features. This process is expressed as:

(14) $$\begin{array}{cc} \; & {\overset{ˇ}{F}}_{s}={\tilde{F}}_{s}⊗{\tilde{F}}_{w},{\overset{ˇ}{F}}_{f}={\tilde{F}}_{f}⊗{\tilde{F}}_{w},\\ \; & {\overset{ˇ}{F}}_{s}^{a}=GAP\left({\overset{ˇ}{F}}_{s}\right),{\overset{ˇ}{F}}_{s}^{m}=GMP\left({\overset{ˇ}{F}}_{s}\right),\\ \; & {\overset{ˇ}{F}}_{f}^{a}=GAP\left({\overset{ˇ}{F}}_{f}\right),{\overset{ˇ}{F}}_{f}^{m}=GMP\left({\overset{ˇ}{F}}_{f}\right), \end{array}$$

where ${\overset{ˇ}{F}}_{s}^{a}$, ${\overset{ˇ}{F}}_{s}^{m}$, ${\overset{ˇ}{F}}_{f}^{a}$, ${\overset{ˇ}{F}}_{f}^{m}\in R^{H\times W\times 1}$ denote the pooled spatial features. For each branch, the two paired pooled features are concatenated along the channel dimension. We then adopt a compact CNN architecture to generate spatial attention weight maps. Specifically, this network first utilizes a 3×3 convolution followed by ReLU activation to capture local spatial features. Afterwards, another 3×3 convolution layer is employed to refine spatial feature representations. We sequentially apply normalization and the sigmoid function to produce the ultimate spatial attention weights. This process is defined as:

(15) $$\begin{array}{cc} \; & {\overset{ˇ}{F}}_{s}^{c}=Concat\left({\overset{ˇ}{F}}_{s}^{a},{\overset{ˇ}{F}}_{s}^{m}\right),{\overset{ˇ}{F}}_{f}^{c}=Concat\left({\overset{ˇ}{F}}_{f}^{a},{\overset{ˇ}{F}}_{f}^{m}\right),\\ \; & {\overset{ˇ}{F}}_{s}^{cl}=CNN\left({\overset{ˇ}{F}}_{s}^{c}\right),{\overset{ˇ}{F}}_{f}^{cl}=CNN\left({\overset{ˇ}{F}}_{f}^{c}\right), \end{array}$$

where ${\overset{ˇ}{F}}_{s}^{cl}$, ${\overset{ˇ}{F}}_{f}^{cl}\in R^{H\times W\times 1}$ represent the spatial attention weight maps of the two branches. These weight maps are element-wise multiplied with the corresponding original features to generate spatially enhanced features ${\overset{ˇ}{F}}_{s}^{se}$ and ${\overset{ˇ}{F}}_{f}^{fe}$. Finally, the spatially enhanced features are added to the original inputs ${\tilde{F}}_{s}$ and ${\tilde{F}}_{f}$ to obtain the final optimized feature representations. This procedure is formulated as:

(16) $$\begin{array}{cc} \; & {\overset{ˇ}{F}}_{s}^{se}={\overset{ˇ}{F}}_{s}^{cl}⊗{\tilde{F}}_{s},{\overset{ˇ}{F}}_{f}^{fe}={\overset{ˇ}{F}}_{f}^{cl}⊗{\tilde{F}}_{f},\\ \; & {\hat{F}}_{s}={\overset{ˇ}{F}}_{s}^{se}+{\tilde{F}}_{s},{\hat{F}}_{f}={\overset{ˇ}{F}}_{f}^{fe}+{\tilde{F}}_{f}, \end{array}$$

where ${\hat{F}}_{s}$ and ${\hat{F}}_{f}$ denote the final output of the BCMU.

### 3.4. Hybrid Extraction Block

The proposed MFMEU, FCPMU, and BCMU collectively form a core feature extraction unit, namely the hybrid extraction block (HEB). This block implements deep feature interaction and adaptive feature enhancement via a dual-branch parallel structure coupled with bidirectional cross modulation. The overall process of the HEB is defined as:

(17) $$\begin{array}{cc} \; & {\tilde{F}}_{s}=MFMEU\left(F_{s}\right),{\tilde{F}}_{f}=FCPMU\left(F_{f}\right),\\ \; & {\hat{F}}_{s},{\hat{F}}_{f}=BCMU\left({\tilde{F}}_{s},{\tilde{F}}_{f}\right), \end{array}$$

where $F_{s}$, ${\tilde{F}}_{s}$, $F_{f}$, ${\tilde{F}}_{f}$, ${\hat{F}}_{s}$ and ${\hat{F}}_{f}$ follow the same definitions as provided above.

## 4. Experimental Results

### 4.1. Datasets and Implementation Details

**Datasets.** During our experiments, we adopt CVC-09-1K as the training set, containing 1000 infrared images randomly sampled from the FIR Sequence Pedestrian Dataset (CVC-09) [34]. This dataset covers diverse urban scenes with pedestrians, roads, vegetation and buildings, and all images share a fixed resolution of 640×480. Low-resolution (LR) samples are generated by bicubic downsampling of corresponding high-resolution (HR) images. For evaluation, we use resultsA [58], resultsC [59], M3FD [60], IR700 [61], and FLIR [62] as test datasets. These test datasets cover diverse acquisition scenarios, including urban buildings, road traffic, visible-infrared pairs, outdoor landscapes, and automotive infrared scenes. To ensure rigorous evaluation, we perform comprehensive image deduplication and scene screening to remove highly similar samples across training and test datasets. We utilize peak signal-to-noise ratio (PSNR) and structural similarity index measure (SSIM) as quantitative metrics, with results averaged over all image channels. Apart from quantitative and qualitative evaluations under standard bicubic degradation, we further evaluate model robustness against simulated real degradations in Section 4.3.

**Implementation details.** During training, we randomly crop 16 patches of size 64×64 from LR input, followed by standard data augmentation strategies including random horizontal flipping and rotation. The baseline of the proposed DMMN is constructed with 8 HEBs and 36 feature channels. The model is optimized for 100,000 iterations using the Adam optimizer [63] ($\beta_{1}=0.9$, $\beta_{2}=0.99$). The initial learning rate is set to $1\times 10^{-3}$, decayed by a factor of 0.5 every 25,000 iterations via the MultiStepLR [64]. All experiments are implemented using PyTorch 1.13.0 and CUDA 11.8 on a Linux workstation equipped with an NVIDIA RTX 3090 GPU (24 GB VRAM), an Intel Xeon E5-2620 v4 CPU, 32 GB of system memory.

To validate the statistical stability of the quantitative results, we adopt standardized repeated evaluation protocols. Two independent training runs are conducted with fixed random seeds (51 and 61) under identical configurations, and the reported PSNR and SSIM results are averaged over the two testings across all five infrared test datasets. The PSNR fluctuation between experiments is within 0.02 dB, verifying that our performance gains of 0.01 dB to 0.08 dB are statistically stable. Moreover, we measure inference latency and memory consumption three times for each test dataset on 50 test images following a unified timing protocol, and the averaged results are used to exclude random GPU cache fluctuations. For fair comparison, all visible SR methods [7,9,10,13,14,15,16,41,65] are retrained under consistent experimental settings, while infrared SR methods [25,30,31,50,53] are evaluated using their official pre-trained weights within our unified testing.

### 4.2. Comparisons with State-of-the-Art Methods

**Quantitative comparison.** To evaluate the proposed DMMN, we compare it with 14 lightweight SR methods, including nine visible methods: EDSR [7], RFDN [9], ShuffleMixer [41], HNCT [65], SwinIR-light [13], SAFMN [10], SRFormer-light [14], HiT-SR [16], CATANet [15], and five infrared methods: PSRGAN [25], RLKA-Net [50], LIRSRN [53], CoRPLE [31], and DifIISR [30]. Quantitative results for ×2 and ×4 upscaling are summarized in Table 1. Apart from conventional reconstruction metrics (i.e., PSNR and SSIM), we report model complexity including parameter counts (#Params) and floating point operations (#FLOPs). For fair evaluation, the complexity statistics are calculated via fvcore.nn.flop_count_str https://detectron2.readthedocs.io/en/latest/modules/fvcore.html#fvcore.nn.parameter_count (accessed on 11 April 2026) under a unified setting, where LR images are upscaled to 1280×960 pixels.

Benefiting from its efficient design, the proposed DMMN attains competitive reconstruction results across five infrared benchmark datasets, with merely 386 K parameters and 19 G FLOPs. For ×4 SR tasks, DMMN surpasses SwinIR-light [13] by 0.09 dB in PSNR on the FLIR dataset, while its parameters and FLOPs are only 41.5% and 22.9% of SwinIR-light, respectively. Compared with CoRPLE [31], DMMN obtains consistently better overall results and drastically cuts parameters and FLOPs down to 6.1% and 7.2% of CoRPLE. Under the ×2 SR, DMMN outperforms SRFormer-light [14] across all test datasets, with around 55.8% fewer parameters and 75.9% lower FLOPs. These results demonstrate that the proposed DMMN achieves a favorable trade-off between reconstruction accuracy and computational complexity.

**Figure 3:**
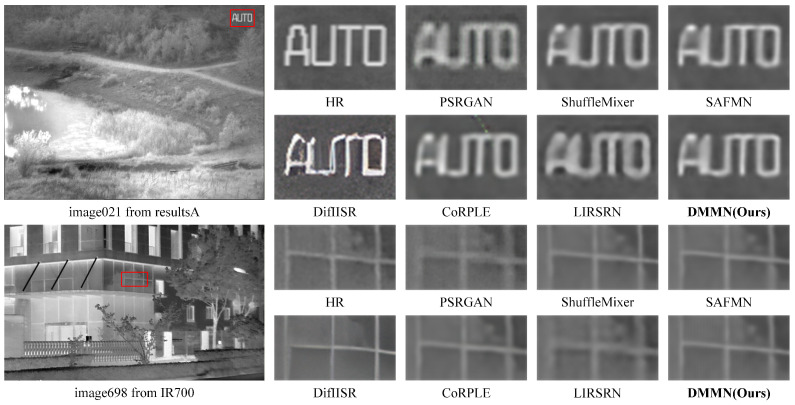
Visual comparison of different methods for ×4 SR. The proposed DMMN restores a more accurate image structure.

**Qualitative comparisons.** To intuitively show texture recovery differences across mainstream methods, we select typical lightweight models covering CNN-based method [41], ViT-like approach [10] and dedicated IRSR methods [25,30,31,53] for visual comparison. We present visual comparisons of the proposed DMMN and lightweight-based methods for ×4 SR. As illustrated in Figure 3, most competing methods suffer from blurring artifacts and fail to recover faithfully texture details, while our DMMN reconstructs more accurate textures and structures. For the text sample, DifIISR [30] produces obvious character distortions (e.g., “T” is wrongly reconstructed as “Γ”). PSRGAN [25], ShuffleMixer [41] and LIRSRN [53] yield blurry edges, and SAFMN [10] and CoRPLE [31] exhibit insufficient edge sharpness. By contrast, DMMN produces distinct outlines and sharp character boundaries. In the grid-structured scene, the reconstruction results of most methods [10,25,41,53] suffer from obvious edge blurring, while DMMN restores continuous, delicate grid lines.

**Memory usage and running time comparisons.** To evaluate the efficiency of the proposed DMMN, we compare the GPU memory consumption (#GPU.Mem) and average inference time (#Avg.Time) of different methods under identical settings, as listed in Table 2. We adopt 50 LR infrared images of size 160×120 as inputs, which are reconstructed to 640×480 HR outputs for ×4 SR. Prior to timing measurement, we execute five warm-up forward passes to eliminate GPU cache and initialization overhead. The batch size is set to 1 to simulate single-image inference on embedded devices. We adopt torch.cuda.synchronize() https://docs.pytorch.ac.cn/docs/1.13/generated/torch.cuda.synchronize.html (accessed on 15 April 2026) to exclude latency induced by CUDA asynchronous execution before and after each forward pass. For inference time, we perform three times on 50 images and then average them as the final result. Moreover, we compute the peak GPU memory occupation via torch.cuda.max_memory_allocated() https://docs.pytorch.org/docs/1.13/generated/torch.cuda.max_memory_allocated.html (accessed on 15 April 2026).

To evaluate the inference efficiency comprehensively without redundant data, we select representative lightweight models covering three mainstream categories. These selected baselines include classic CNN lightweight models [41,65], Transformer architectures [13,16], and dedicated IRSR methods [25,31,50] to demonstrate the efficiency advantages of our DMMN. In terms of inference time, the proposed DMMN achieves 43.87 ms, representing around a 44.2% reduction compared with higher-performance CoRPLE [31]. For GPU memory consumption, DMMN reduces resource usage by 42.0% compared with the window-based SwinIR-light [13]. Compared to HiT-SR [16], which uses hierarchical window strategy to efficiently aggregate hierarchical features, the DMMN is ×2.3 times faster. Combined with the above Table 1 and Figure 3, these results demonstrate our DMMN achieving a competitive balance between reconstruction accuracy and computational overhead.

**Figure 4:**
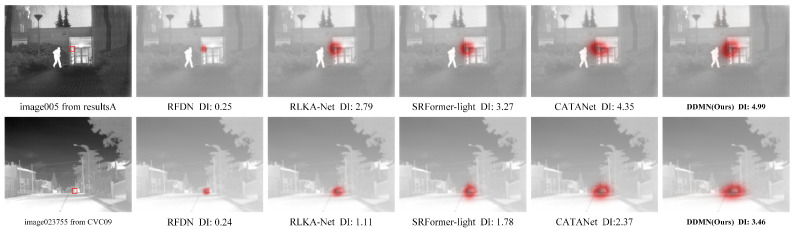
Comparison of LAM results among different methods in ×4 infrared image super-resolution. When reconstructing the image patch marked by the red box, the red regions indicate the effective pixels used for reconstruction. Darker colors indicate higher contributions. The diffusion index (DI) reflects the range of pixels involved in image reconstruction. A higher DI value indicates broader coverage and the involvement of more pixels. The results show that our method has a larger receptive field and can engage more pixels for image reconstruction.

**LAM comparisons.** The local attribution map (LAM) [66] reflects the strong correlation between red-highlighted pixels and the target rectangular patch during reconstruction, while the diffusion index (DI) [66] quantifies the scope of feature interactions. A higher DI corresponds to a broader attention scope. As illustrated in Figure 4, RFDN [9] only covers a limited spatial region. While RLKA-Net [50], SRFormer-light [14], and CATANet [15] yield gradually expanded attention areas, their activations lack sufficient correlation with the global structure of target regions. By comparison, the proposed DMMN produces a wider LAM activation range. These results verify that our DMMN can efficiently exploit non-local information for efficient IRSR.

### 4.3. Cross-Scene Visualization Comparison

**Figure 5:**
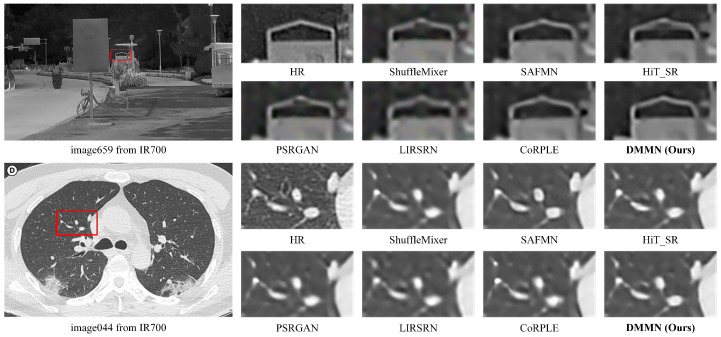
Visual comparisons under Gaussian noise (σ = 4) and on medical infrared images. The upper row indicates that our DMMN effectively suppresses noise and recovers finer structural textures. The second row reveals that the proposed method maintains smooth background regions while preserving intact lesion boundaries. These observations verify the strong cross-domain generalization of our model under complex degradation conditions.

The above quantitative and visual comparisons on standard bicubic degradation verify the efficiency and reconstruction performance of DMMN. To further validate the model’s practical robustness against real sensor noise and cross-domain data, we conduct cross-scene visualization tests under complex degradation. We present two groups of cross-scene visual comparisons for ×4 SR in Figure 5. The upper row of Figure 5 exhibits reconstruction results under bicubic downsampling plus additive Gaussian noise (σ = 4), simulating noise interference from practical infrared sensors. The lower row displays real medical images reconstruction results, which are characterized by inherent uneven illumination and blurry soft tissue. As observed in Figure 5, the reconstruction results of ShuffleMixer [41], SAFMN [10], HiT-SR [16], PSRGAN [25] and LIRSRN [53] suffer from obvious edge blurring and texture degradation on noisy inputs. CoRPLE [31] can recover coarse outlines yet struggles to preserve small structural details. In contrast, the proposed DMMN effectively reconstructs clear edges and fine structure textures.

### 4.4. Ablation Study

In this section, we perform multiple ablation experiments to evaluate the contribution of each component within DMMN. All experiments adopt the ×4 DMMN configuration and are trained on the CVC-09-1K dataset [34] for fair comparisons. Quantitative results on the resultsA [58] and resultsC [59] datasets are summarized in Table 3, Table 4, Table 5 and Table 6.

**Effectiveness of multi-scale feature modulation enhancement unit.** To verify the effectiveness of MFMEU, we conduct ablation experiments built upon the DMMN baseline. As presented in Table 3, replacing MFMEU with FCPMU yields an average drop of 0.02 dB in PSNR over the two datasets, which confirms the positive contribution of MFMEU. Removing either branch within MFMEU results in performance degradation, demonstrating that the dual-branch design achieves synergistic performance improvements. Specifically, eliminating the multi-scale feature modulation branch leads to an average reduction of 0.045 dB in PSNR across two test datasets. These results confirm the importance of MFMEU. Accordingly, we further analyze this module to explore its working mechanism.

- Multi-scale representation. We conduct ablation studies on multi-scale feature representation to explore its role within MFMEU. As listed in Table 3, removing this operation leads to an average PSNR drop of 0.02 dB on the resultsA and resultsC datasets, whereas FLOPs rise by about 5.0% and inference time increases by around 0.5%. Furthermore, we replace the original pooling operation with adaptive average pooling and nearest interpolation for discriminative feature extraction. Ablation experiments show the parameter count remains unchanged and computational overhead and latency both increase, accompanied by performance degradation. These findings demonstrate that multi-scale representation built upon adaptive max pooling contributes to improving reconstruction quality.
- Feature Modulation. Feature modulation is introduced to endow the network with adaptive representation capacity. Table 3 shows that removing this component reduces the average PSNR by 0.02 dB on the resultsA and resultsC datasets. When both multi-scale feature representation and feature modulation are removed, the average PSNR declines by 0.03 dB on the two datasets, and the inference time rises by approximately 20.81%.

**Figure 6:**
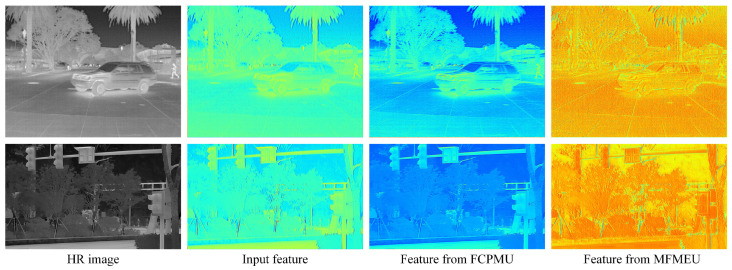
Visualization of feature maps generated by the core modules FCPMU and MFMEU. The more orange yellow, the richer the edge and texture details of the image; The more blue-green, the smoother and more regular the global contour structure. FCPMU tends to extract low-frequency global structural features, while MFMEU focuses on capturing high-frequency local detail features.

**Effectiveness of frequency-domain cross-correlation patch modulation unit.** To verify the effectiveness of the FCPMU module, we conduct multiple ablation experiments. As shown in Table 4, replacing FCPMU with MFMEU reduces the PSNR by 0.10 dB on the resultsA dataset and 0.13 dB on the resultsC dataset while increasing the average inference time by 10.1%. These results demonstrate the essential role of FCPMU in our model. We further explore the internal factors that account for its superior performance. In addition, Figure 6 visualizes the feature maps generated by FCPMU and MFMEU. The visualization results show that FCPMU produces uniform responses to the overall contours and background features of infrared images, whereas MFMEU yields stronger activation in fine-grained regions, including edges and textures.

- Frequency-domain operation. We efficiently capture global features by incorporating frequency-domain operations. Although removing the FFT module reduces the inference time by 1.33 ms, it degrades reconstruction performance, resulting in a PSNR drop of 0.04 dB on resultsA and 0.03 dB on resultsC. These results demonstrate that frequency-domain operations enable the model to exploit long-range dependencies and enrich global feature representations for IRSR.
- Patch operation. Removing the patch operation (i.e., “w/o Patch operation”) causes noticeable performance degradation and higher inference latency. Increasing or decreasing the patch size from the default value of 8 to 4 or 12 leads to overall performance declines, and the runtime increases by 22.8% and 7.8%, respectively. These findings reveal that an appropriate patch size achieves a favorable trade-off between reconstruction accuracy and computational efficiency.
- Self-attention effect. Replacing the modulation component within FCPMU with SA induces performance degradation and increases inference latency by 7.8%. Furthermore, substituting FCPMU with window-based multi-head self-attention (W-MSA) yields moderate performance gains yet substantially elevates FLOPs and runtime by 10.6% and 51.7%, respectively. The above results verify that leveraging frequency-domain operations enables FCPMU to maintain favorable reconstruction quality with reduced computational demands.

**Effectiveness of bidirectional cross modulation unit.** We conduct ablation experiments by removing or substituting internal components to verify the effectiveness of BCMU. As illustrated in Table 5, removing BCMU reduces GPU memory consumption and inference latency yet triggers substantial degradation of reconstruction performance. For example, the PSNR on the resultsC dataset declines by 0.14 dB. Eliminating channel attention, spatial attention, or the cross-operation inside BCMU independently leads to performance losses. In addition, swapping the order of attention branches by placing spatial attention ahead of channel attention yields significant performance drops and lower inference efficiency. Specifically, the PSNR decreases by 0.11 dB on the resultsA dataset and 0.13 dB on the resultsC dataset, while the runtime rises by approximately 3.0%. These results reveal that although BCMU introduces moderate computational overhead, it notably boosts reconstruction accuracy without sacrificing favorable inference efficiency.

**Effectiveness of FFTloss.** To evaluate the effectiveness of the frequency-domain loss, we conduct ablation experiments from two dimensions including whether to introduce FFT loss and the selection of different weight values of λ in Table 6. Removing FFT loss does not alter model parameters, FLOPs or memory consumption, yet inference time rises by approximately 1.34% and reconstruction performance undergoes obvious degradation. For instance, the average PSNR drops by 0.10 dB on the resultsA and resultsC datasets. Further ablation on the weight of FFT loss indicates that overly small or large weight coefficients lead to degraded reconstruction performance. These results demonstrate that the frequency-domain loss can boost reconstruction quality, and appropriate weight setting is essential to fully unleash its performance.

## 5. Conclusions

In this paper, we propose a simple yet effective DMMN for efficient infrared image super-resolution. The core of DMMN consists of the synergistic design of MFMEU and FCPMU, which jointly capture cross-scale spatial details and global feature representations from complementary perspectives. Moreover, we develop a BCMU to facilitate feature interaction between outputs from the MFMEU and FCPMU via sequential channel-wise and spatial-wise cross-attention. Extensive experimental results demonstrate that the proposed DMMN achieves a competitive trade-off between reconstruction performance and computational efficiency. Although DMMN achieves favorable performance with low computational complexity, further research is still required to handle extreme super-resolution tasks, including ×8 and larger upscaling factors, as well as complex real-world degradation scenarios. Future work will optimize the feature extraction and modulation mechanisms to adapt to ×8 and more extreme scenarios. We also plan to incorporate a motion perception module to strengthen model robustness against dynamic scenes with motion blur.

## Figures and Tables

**Table 1:** Comparative results of lightweight SR methods on five benchmark datasets. The reported PSNR and SSIM values are averaged over all image channels. The number of FLOPs is measured for high-resolution output of a size 1280×960 pixels. The PSNR/SSIM results of visible methods and our DMMN are averaged from two independent training runs with identical hyperparameters. The top two methods are highlighted in red and blue, respectively.

Scale	Model	#Params [K]	#FLOPs [G]	resultsA	resultsC	M3FD	IR700	FLIR
×2	EDSR [7]	1370	422	38.3814/0.9392	39.3267/0.9504	38.8992/0.9620	36.1400/0.9255	34.7218/0.8158
	RFDN [9]	417	122	38.3906/0.9393	39.3129/0.9505	38.8055/0.9617	35.9243/0.9229	34.7596/0.8160
	PSRGAN [25]	313	-	36.8919/0.9191	37.8214/0.9332	36.8629/0.9441	34.0270/0.8921	33.3451/0.7904
	ShuffleMixer [41]	394	121	38.4335/0.9391	39.3010/0.9503	38.8462/0.9620	35.9939/0.9249	34.7170/0.8150
	HNCT [65]	357	111	38.4383/0.9389	39.3424/0.9504	38.9623/0.9627	36.0086/0.9239	34.8663/0.8168
	SwinIR-light [13]	910	326	38.4931/0.9399	39.3779/0.9509	39.0246/0.9632	36.2425/0.9283	35.0459/0.8191
	SAFMN [10]	228	69	37.7929/0.9397	39.3614/0.9511	38.9309/0.9627	36.0355/0.9268	34.7363/0.8152
	RLKA-Net [50]	225	253	37.9233/0.9309	39.0137/0.9448	38.7270/0.9600	35.2955/0.9041	34.9281/0.8164
	SRFormer-light [14]	853	308	38.4783/0.9393	39.3894/0.9508	39.0101/0.9625	36.2345/0.9263	35.1047/0.8221
	HiT-SR [16]	847	299	38.4169/0.9392	39.3338/0.9504	38.8963/0.9622	35.8704/0.9224	35.1065/0.8226
	CATANet [15]	477	197	38.4555/0.9397	39.3809/0.9511	38.9570/0.9626	36.1062/0.9254	35.1209/0.8231
	LIRSRN [53]	49	14	38.0847/0.9392	38.7952/0.9503	38.1370/0.9606	35.7103/0.9239	33.7941/0.7949
	CoRPLE [31]	6.2 M	785	37.8686/0.9355	38.7100/0.9475	37.5546/0.9560	34.9269/0.9178	34.8464/0.8155
	DifIISR [30]	-	-	33.7641/0.8448	34.1549/0.8593	31.9463/0.8534	30.6968/0.8177	31.4826/0.6979
	DMMN(Ours)	374	71	38.4780/0.9410	39.3939/0.9523	39.0317/0.9638	36.3523/0.9292	35.1049/0.8234
×4	EDSR [7]	1158	152	33.3254/0.8373	33.9173/0.8543	31.6399/0.8459	30.2482/0.8104	31.3399/0.6934
	RFDN [9]	433	32	33.4022/0.8388	34.0277/0.8560	31.7628/0.8489	30.3170/0.8123	31.4287/0.6951
	PSRGAN [25]	350	-	32.0117/0.8084	32.7532/0.8284	30.3637/0.8131	28.8120/0.7467	30.5874/0.5983
	ShuffleMixer [41]	411	37	33.4013/0.8395	34.0237/0.8574	31.6698/0.8485	30.2232/0.8107	31.4103/0.6949
	HNCT [65]	373	29	33.4803/0.8410	34.1447/0.8587	31.8265/0.8513	30.3152/0.8128	31.4730/0.6968
	SwinIR-light [13]	930	83	33.4872/0.8402	34.1788/0.8590	31.8634/0.8501	30.4047/0.8143	31.4388/0.6938
	SAFMN [10]	240	18	33.3886/0.8400	34.0166/0.8576	31.7708/0.8506	30.2877/0.8122	31.4790/0.6968
	RLKA-Net [50]	245	65	33.4368/0.8383	34.0661/0.8552	31.8182/0.8502	30.2290/0.8112	31.4211/0.6945
	SRFormer-light [14]	873	79	33.4576/0.8400	34.1066/0.8571	31.8100/0.8484	30.4203/0.8144	31.4653/0.6954
	HiT-SR [16]	866	77	33.4082/0.8385	34.0121/0.8556	31.7489/0.8485	30.3081/0.8111	31.4033/0.6940
	CATANet [15]	535	64	33.4915/0.8406	34.1343/0.8582	31.8451/0.8506	30.2974/0.8124	31.4481/0.6954
	LIRSRN [53]	70	5	32.8769/0.8349	33.4085/0.8524	31.0894/0.8396	29.8565/0.7973	30.5096/0.6245
	CoRPLE [31]	6.3 M	264	33.5079/0.8413	34.0914/0.8581	31.9074/0.8518	30.3768/0.8147	31.4426/0.6958
	DifIISR [30]	-	-	27.7673/0.6464	28.4338/0.6664	27.5891/0.7156	28.0799/0.7209	29.2579/0.6106
	DMMN(Ours)	386	19	33.5291/0.8421	34.1673/0.8597	31.9553/0.8538	30.4929/0.8154	31.5264/0.6980

**Table 2:** Comparison of memory usage and running time of lightweight models for ×4 SR. #GPU.Mem. represents the maximum GPU memory allocated during inference, measured using the torch.cuda.max_memory_allocated() https://docs.pytorch.org/docs/1.13/generated/torch.cuda.max_memory_allocated.html (accessed on 15 April 2026) function. #Avg.Time. denotes the average inference time across 50 LR images of size 160×120 pixels. Inference time and peak GPU memory consumption are measured over three repeated full test cycles and reported as averaged values. The top-two methods are highlighted in red and blue, respectively.

		Quality Metrics	
Type	Methods	#GPU.Mem. [M]	#Avg.Time. [ms]
different lightweight SR methods	ShuffleMixer	228.25	24.33
	HNCT [65]	116.02	59.13
	SwinIR-light [13]	265.53	111.07
	HiT-SR [16]	1231.25	101.05
	PSRGAN [25]	165.08	56.30
	RLKA-Net [50]	46.12	53.60
	CoRPLE [31]	635.41	78.64
	DMMN(Ours)	153.89	43.87

**Table 3:** Ablation study of MFMEU on the resultsA and resultsC datasets for ×4 SR. “A → B” represents replacing component A with component B. “w/o C” denotes removing component C. The baseline DMMN is shown in bold black.

Ablation	Variant	#Params [K]	#FLOPs [G]	#GPU.Mem. [M]	#Avg.Time. [ms]	resultsA	resultsC
MFMEU	MFMEU → FCPMU	400	18.57	153.79	42.45	33.5089/0.8415	34.1464/0.8590
	w/o MFM branch	340	16.11	153.74	42.27	33.4824/0.8413	34.1262/0.8590
	w/o LFE branch	317	13.36	153.61	42.64	33.5005/0.8411	34.1437/0.8589
	(a): w/o Multi-scale Representation	386	19.51	153.89	44.09	33.5081/0.8412	34.1445/0.8591
	(b): w/o Feature Modulation	386	18.56	153.89	44.39	33.5136/0.8420	34.1490/0.8596
	(a) + (b)	386	19.51	153.89	52.99	33.5005/0.8409	34.1368/0.8590
	AdaptiveMaxPool → AdaptiveAvgPool	386	18.56	153.89	47.87	33.5018/0.8419	34.1361/0.8594
	AdaptiveMaxPool → Nearest interpolate	386	18.54	153.89	44.59	33.5242/0.8418	34.1699/0.8595
Baseline	**DMMN**	**386**	**18.53**	**153.89**	**43.87**	**33.5291**/**0.8421**	**34.1673**/**0.8597**

**Table 4:** Ablation study of FCPMU on the resultsA and resultsC datasets for ×4 SR. “A → B” and “w/o C” have the same meaning as Table 3. The baseline DMMN is highlighted in bold black.

Ablation	Variant	#Params [K]	#FLOPs [G]	#GPU.Mem. [M]	#Avg.Time. [ms]	resultsA	resultsC
FCPMU	FCPMU → MFMEU	371	18.50	153.63	48.32	33.4303/0.8410	34.0392/0.8587
	w/o FFT operation	386	18.53	153.89	42.54	33.4934/0.8414	34.1379/0.8590
	w/o Patch operation	389	18.53	153.73	44.48	33.5091/0.8411	34.1605/0.8589
	Patchsize = 4	386	18.53	153.89	53.86	33.4844/0.8420	34.1216/0.8597
	Patchsize = 12	386	18.63	153.71	47.29	33.5269/0.8421	34.1644/0.8596
	FFT Modulation → Self-Attention	389	18.53	153.73	47.31	33.5064/0.8411	34.1633/0.8589
	FCPMU → W-MSA	383	20.50	153.98	66.54	33.5352/0.8421	34.1898/0.8597
Baseline	**DMMN**	**386**	**18.53**	**153.89**	**43.87**	**33.5291**/**0.8421**	**34.1673**/**0.8597**

**Table 5:** Ablation study of BCMU on the resultsA and resultsC datasets for ×4 SR. “w/o C” has the same meaning as Table 3. The baseline DMMN is shown in bold black.

Ablation	Variant	#Params [K]	#FLOPs [G]	#GPU.Mem. [M]	#Avg.Time. [ms]	resultsA	resultsC
BCMU	w/o BCMU	237	16.91	74.09	22.28	33.4239/0.8420	34.0272/0.8595
	w/o Channel Attention (CA)	386	19.33	82.84	35.00	33.5087/0.8409	34.1457/0.8586
	w/o Spatial Attention (SA)	386	18.50	153.71	39.75	33.4382/0.8403	34.0739/0.8582
	w/o Cross operation	386	18.53	153.89	44.82	33.5202/0.8420	34.1602/0.8597
	SA then CA	386	18.53	159.13	45.16	33.4197/0.8403	34.0378/0.8579
Baseline	**DMMN**	**386**	**18.53**	**153.89**	**43.87**	**33.5291**/**0.8421**	**34.1673**/**0.8597**

**Table 6:** Ablation study of FFTloss on the resultsA and resultsC datasets for ×4 SR. The baseline DMMN is highlighted in bold black. “✓” represents the use of corresponding weight coefficients.

λ					#Params [K]	#FLOPs [G]	#GPU Mem. [M]	#Avg.Time [ms]	resultsA	resultsC
0.0	0.05	0.1	0.5	1.0						
✓					386	18.53	153.89	44.46	33.4357/0.8416	34.0648/0.8593
	✓				386	18.53	153.89	44.17	33.5021/0.8421	34.1257/0.8596
		✓			386	18.53	153.89	44.99	33.5222/0.8420	34.1613/0.8596
				✓	386	18.53	153.89	45.55	33.5161/0.8420	34.1539/0.8597
			✓		**386**	**18.53**	**153.89**	**43.87**	**33.5291**/**0.8421**	**34.1673**/**0.8597**

## Data Availability

The Data are openly available in a public repository. The code is available at https://github.com/smilenorth1/DMMN-main (accessed on 1 August 2026).
